# Immersive Surgical Anatomy of the Craniometric Points

**DOI:** 10.7759/cureus.8643

**Published:** 2020-06-15

**Authors:** Vera Vigo, Kimberly Cornejo, Lizbeth Nunez, Adib Abla, Roberto Rodriguez Rubio

**Affiliations:** 1 Neurological Surgery, University of California, San Francisco, USA

**Keywords:** craniometric points, cerebral cortex, vascular landmarks, ventricular access, keyholes, motor cortex, speech area, volumetric models, cranial sutures

## Abstract

Craniometric points (CPs) have been used in neurosciences since the 1800s. Localization of the CPs allows for the identification of crucial intracranial structures. Despite the contribution of advanced technology to surgery, the knowledge of these points remains crucial for surgical planning and intraoperative orientation. The understanding of these crucial points can be facilitated with the use of three-dimensional technology combined with anatomical dissections. The present study is part of a stereoscopic collection of volumetric models (VMs) obtained from cadaveric dissections that depict the relevant anatomy of the CPs. Five embalmed heads and two dry skulls have been used to depict these points. After the anatomical dissection, stereoscopic images and VMs were generated to show the correlation between external and internal landmarks. The CPs identified were divided into sutures, suture junctions, prominences and depressions, and cortical surface landmarks. The VMs represent an interactive way to define these points easily and their correlation with different intracranial structures (vascular structure, ventricle cavity, and Brodmann’s areas).

## Introduction

Craniometry is a science that utilizes measurements of the skull and facial structures with the aim of analysing specific osseous features in different populations. Therefore, craniometric points (CPs) were described as the landmarks from which these measurements could be taken. The CPs have been used for several purposes in anthropology, forensic sciences, and neurosciences. In 1876, Broca published the “Sur la topographie crânio-cérébrale” and, for the first time, correlated the CPs to intracranial structures such as sulci and gyri of interest [1]. In the following years, anatomists, surgeons, and radiologists have studied the craniocerebral topography and measured lines from different CPs to identify them easily. In the late 19th century, the application of the CPs in surgery established the foundation of modern neurosurgery, with the possibility to tailor craniotomies in specific areas of interest [1]. Recently, Ribas and others have focused most of their anatomical research around the surgical applications of CPs, underlying their importance in modern neurosurgery [1-4].

Nowadays, preoperative and intraoperative surgical planning is based on neuroimaging information. With the advent of neuronavigation systems, the recognition of critical intracranial structures has been simplified. Nevertheless, such a sophisticated technology cannot fully replace the three-dimensional (3D) understanding of the craniocerebral relationships that can be utilized for the intraoperative assessment of anatomy. The knowledge of these anatomical landmarks should be the starting point of each cranial neurosurgical procedure.

In this study, we will review the available literature related to CPs with the aim of describing their location, relation to critical structures (i.e., cortical areas, vasculature, and ventricles), and their implication for neurosurgical procedures. Moreover, with the use of anatomical volumetric models (VMs) and online 3D platforms, we aimed to facilitate the visuospatial understanding of the CPs and their correlation with intracranial structures.

## Technical report

Materials and methods

Five embalmed and latex-injected cadaveric heads were prepared for anatomical dissections and two dry skulls were utilized to identify the CPs. Dissections were performed under a surgical simulation setting. After removing the scalp and reflecting the temporalis muscles, the cranial sutures and relevant prominences were identified and isolated. After removing the surrounding bone with a high-speed drill (Midas Rex; Medtronic, Minneapolis, MN, USA), the following windows were created: superior frontal, inferior frontal, sphenoid, superior parietal, inferior parietal, temporal, and occipital. The relationship between the CPs and intracranial structures at different layers (i.e., dura, arachnoid, brain surface, white matter, and ventricular) were analysed. Dissections were photo-documented using a professional camera (D810; Nikon, Tokyo, Japan), and selected specimens were reconstructed using 3D data obtained via surface scanning techniques (i.e., photogrammetry and structured light scanning). Our laboratory had previously documented the comprehensive workflow of 3D scanning using the aforementioned techniques [5]. Additionally, the 3D reconstruction of the left brain hemisphere of a healthy male obtained from magnetic resonance was included. The VMs were post-processed using a computer graphics software (Blender 2.82; Blender Foundation, Amsterdam, Holland), and the corresponding texture maps were generated with a 3D texturing program (Substance Painter; Adobe, San Jose, CA, USA).

No IRB/ethics committee approval was required for this study.

Virtual platform

The anatomical VMs were uploaded to a web-based 3D model viewer (Sketchfab; Sketchfab Inc, New York, NY, USA), a platform that belongs to a series of new modalities meant to enhance the immersive and functional capacities of VMs. Once the VMs were uploaded, the virtual scene was prepared for its real-time rendering. Position, lighting, materials, and filters were set to highlight regions of anatomical interest. Strategic points were labeled and annotated for an interactive experience. Views of the models were set for both two-dimensional (2D) and 3D experiences. The stereoscopic version of the virtual scene was set up and tested using a virtual reality headset (HTC Vive; HTC Co., Taiwan, China) and a browser compatible with WebVR technology (Firefox Nightly; Mozilla Co., Mountain View, CA, USA).

Anatomical description

**Figure 1:**
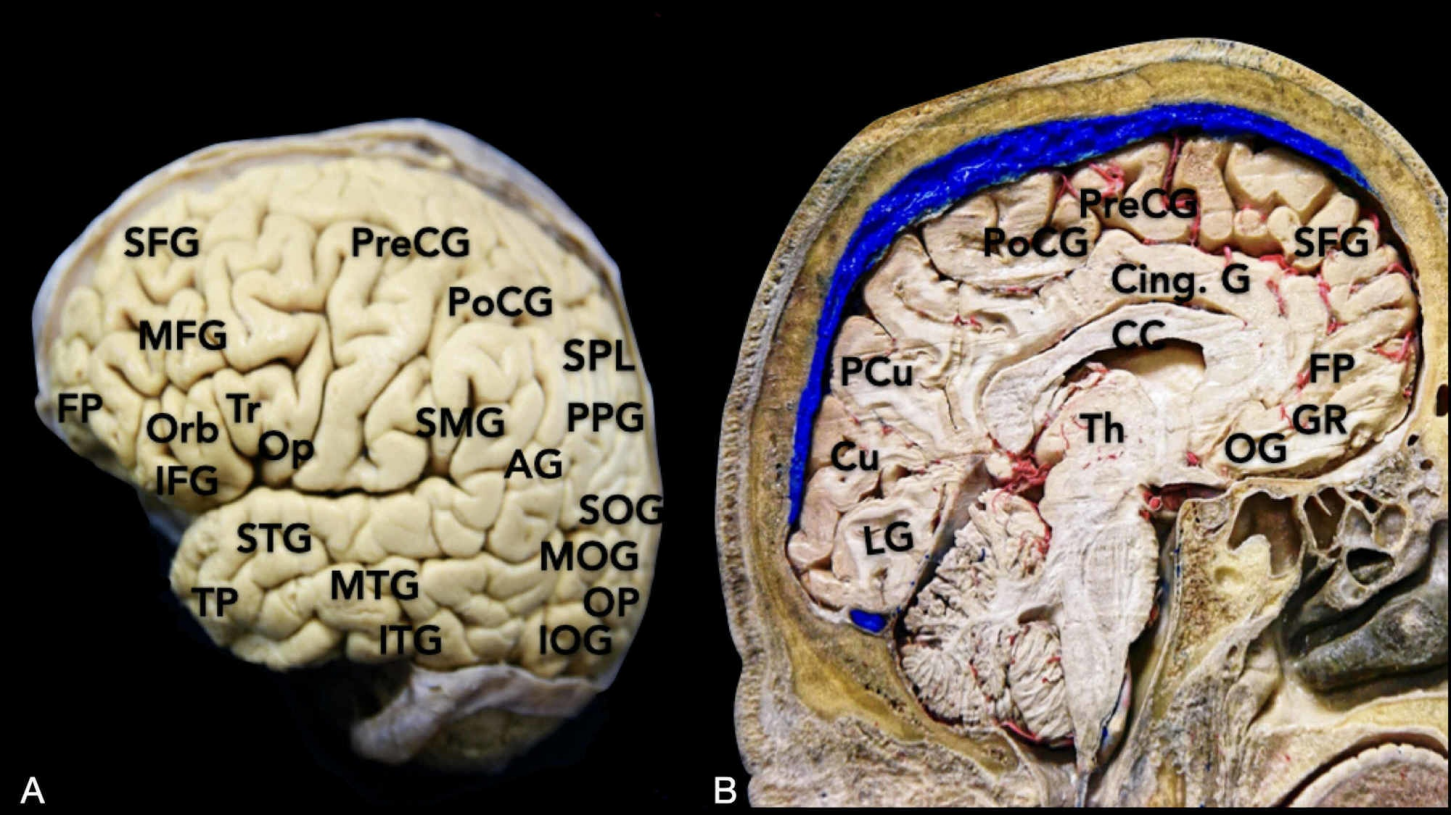
Overview of the main cortical gyri: (A) lateral surface and (B) medial surface of the brain. AG: angular gyrus; CC: corpus callosum; Cing. G: cingular gyrus, Cu: cuneus; FP: frontal pole; GR: gyrus rectus; IFG: inferior frontal gyrus; ITG: inferior temporal gyrus; IOG: inferior occipital gyrus; LG: lingual gyrus; MFG: middle frontal gyrus; MOG: middle occipital gyrus; MTG: middle temporal gyrus; OG: orbital gyrus; Op: pars opercularis of the IFG; Orb: pars orbitalis of the IFG; PCu: precuneus; PoCG: post-central gyrus; PPG: posterior parietal gyrus; PreCG: pre-central gyrus; SFG: superior frontal gyrus; SMG: supramarginal gyrus; SOG: superior occipital gyrus; SPL: superior parietal lobule; STG: superior temporal gyrus; Th: thalamus; TP: temporal pole; Tr: pars triangularis of the IFG.

In the following section, we will review the main osteometric features of the skull including sutures, suture junctions, prominences, and depressions. Afterward, essential cortical points and their relationship with CPs will be reviewed. A clear understanding of the topology of sulci and gyri is fundamental to correlate the intracranial structures with the corresponding superficial landmarks properly (Figure 1, Interactive Model 1).

**Video 1:** Volumetric model of the left hemisphere, with labels of the gyri and annotations of the lateral sulci. Knowledge of the gyri of the brain is critical when performing any brain surgery. This 3D model shows an overview of half of the brain, annotations of the gyri of the lateral, basal, and medial surface. The following instructions can be used to manipulate all models: to move, left click and drag; to zoom in and out, use the mouse scroll. For smartphones and virtual reality (VR)-ready computers, click "view in VR" (glasses icon); to view annotations, click on the numbers, to move around the object, tap or press trigger on the floor using the blinking yellow circle as a pointer. For mobile augmented reality (AR), click on the AR icon (cube) in the top right corner and aim at a horizontal flat surface; once the surface is detected, tap on it to place the model. To view the video in VR mode, Google Cardboard and YouTube mobile app are necessary. First, open the video on the YouTube mobile app and tap the Cardboard icon. Next, place the mobile device inside the Google Cardboard. Finally, look around to view the video in 360°. Quality of the textures and navigation style can be modified by clicking the Settings icon.

Sutures 

**Figure 2:**
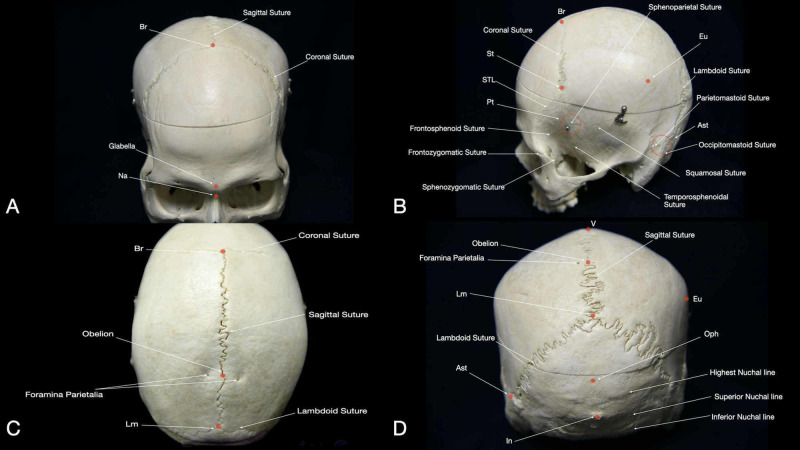
Overview of main sutures, suture junction points, and prominences of the human skull: (A) anterior view, (B) lateral view, (C) superior view, and (D) posterior view. Ast: asterion; Br: bregma; Eu: euryon; In: inion; Lm: lambda; Na: nasion; Oph: ppisthocranium; Pt: pterion; STL: superior temporal line; St: stephanion; V: vertex.

The sutures of the neurocranium are a type of fibrous joint (i.e., synarthroses) that allows the development and expansion of the cranial vault. Around the age of seven years, the ossification of these sutures leads to the formation of the adult skull. Ossification occurs from anterior to posterior and from lateral to medial. These sutures are located between the frontal, parietal, temporal, sphenoid, and occipital bones. The metopic suture is positioned between the two portions of the frontal bone. It is not usually visible in the skull of an adult since its closure occurs in around 9th and 11th months of age [6]. The coronal suture detaches the frontal and parietal bones. This suture forms a subtle depression in the skull that can be commonly located by touch under the scalp (Figure 2A). Over the anterolateral portion of the skull, three main sutures can be found: frontozygomatic, frontosphenoid, and sphenozygomatic (Figure 2B). The frontozygomatic and frontosphenoid sutures merge the frontal bone with the zygomatic and the sphenoid bones, respectively. The sphenozygomatic suture marks the junction of the sphenoid and zygomatic bones on the anterior side of the skull. The sagittal suture is located down the midline of the calvaria convexity connecting the coronal suture and the lambdoidal sutures, thereby, separating the parietal bones (Figure 2C). Laterally, the squamosal suture joins the squamous portion of the temporal bone with the parietal bones and extends horizontally. Towards the inferior posterolateral portion of the skull, three sutures can be found: parietomastoid, occipitomastoid, and lambdoid. The parietomastoid suture separates the parietal bones and the mastoid process of the temporal bone, whereas the occipitomastoid lies at the connection of the inferolateral occipital bone with the posterior portion of the mastoid process. Superior and medial, the lambdoid suture is located at the posterior end of the skull and separates the parietal and the superior portion of the occipital bone (Figure 2D, Interactive Model 2).

**Video 2:** Volumetric model of the skull, with labels of the sutures and the craniometric points of the exocranial and endocranial surfaces.

Suture junctions

**Figure 3:**
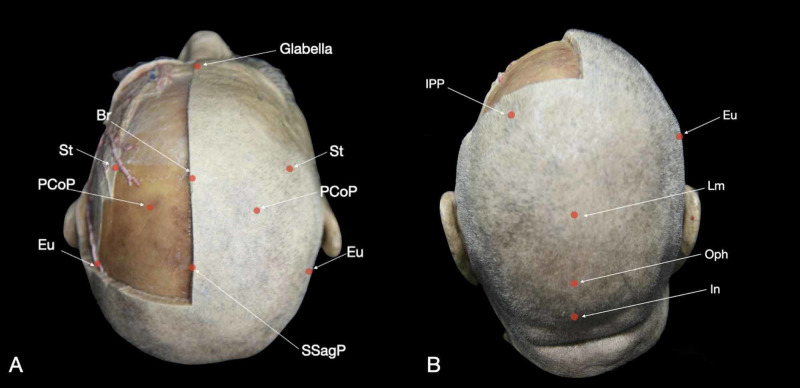
Depiction of the principal craniometric points on the skin: (A) superior view and (B) posterior view of the head. Br: bregma; Eu: euryon; In: inion; IPP: intraparietal point; Lm: lambda; Oph: opisthocranium; PCoP: posterior coronal point; SSagP: superior sagittal point; St: stephanion.

Located at the mid-sagittal plane, bregma (Br) is the point where the coronal suture intersects the sagittal suture (Figures 2A, 3A). This CP corresponds to the anterior fontanelle, which is a diamond-shape membrane-filled space between the frontal and the parietal bones. It is the largest fontanelle and persists until 12-18 months after birth [6]. It is commonly palpated during the neurological examination of newborns to assess intracranial hypertension and to perform ultrasound examination. Its position on the skull makes it a reference point for lateral and anteroposterior coordinates. In adults, the pre-central gyrus is located approximately 4.5 cm posterior to the Br [7]. The point known as the superior Rolandic point (SRP) is found 5 cm posterior to the Br, along the sagittal suture, between the central sulcus (CS) and the interhemispheric fissure (IHF) [8].

**Figure 4:**
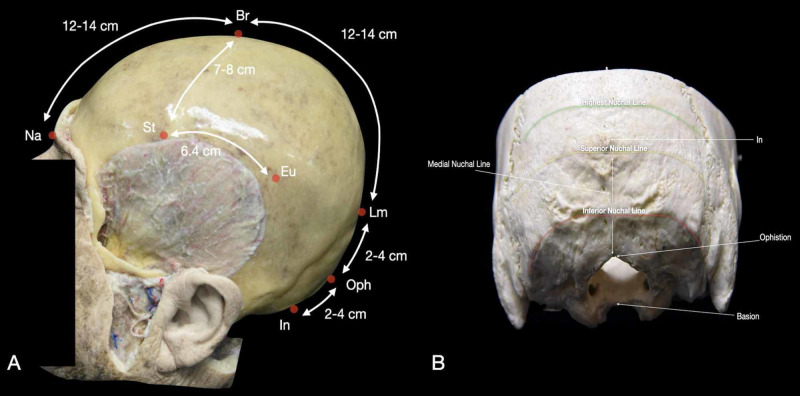
Osteometric points and prominences of the human skull. (A) Lateral view of the skull with the representation of the mean distance of the main craniometric points; (B) posterior view of the skull with an underline of the principal points and prominences. Br: bregma; Eu: euryon; In: inion; Lm: lambda; Na: nasion; Oph: opisthocranium; St: stephanion.

Lambda (Lm) is located at the junction of the lambdoid and sagittal sutures (Figures 2D, 3B). The distance from Br to Lm is about 13 cm, and this measurement runs along the sagittal suture (Interactive Model 3). The distance from nasion to Lm is about 24-26 cm, and it is 2-4 cm superior to the opisthocranion (Figure 4A) [1]. Inferior and lateral to Lm lies the parieto-occipital fissure, which corresponds to the emergence of the parieto-occipital sulcus inside the IHF. The Lm is found 3-5 cm posterior to the obelion; this indicates the intersection of the sagittal suture with the foramina of the parietal emissary veins [9].

**Video 3:** Volumetric model of the skull, with labels of the main craniometric points and their respective lengths.

The pterion (Pt) is an H-shaped sutural junction located between the frontal, parietal, temporal, and sphenoid bones (Figure 2B). The Pt has been classified into four different types depending on how the sutures intersect: sphenoparietal, frontotemporal, stellate, and epipteric (more common in childhood) [10]. The most common is the sphenoparietal type. Pt is an essential landmark for most anterolateral cranial procedures and the pterional craniotomy is centered over this point. Indeed, the Pt stands above the anterior branch of the middle meningeal artery and the Sylvian fissure (SF) (Interactive Model 4). The anterior Sylvian point (ASyP), described below, is located underneath the most posterior portion of the Pt, where the squamous suture intersects the sphenoparietal suture. This point is also known as the anterior squamous point (ASP) [8]. The most anterior and inferior region of the inferior frontal gyrus (IGF) can be located below the Pt (Brodmann's area 44-45, Broca’s area - motor speech programming).

**Video 4:** Volumetric model of a specimen with monolateral cranial windows exposing the dura and annotations of the main craniometric points.

The asterion (Ast), is located laterally and posteriorly at the junction of the lambdoid, parietomastoid, and occipitomastoid sutures (Figure 2B). Two types of Ast have been described based upon the presence of a sutural bone at Ast (Type I) or not (Type II). The second type is the most common [10]. The Ast is a crucial landmark in lateral approaches to the posterior fossa, being the most commonly used in the retrosigmoid approach. In fact, the Ast defines the superior limit of the craniotomy since it serves as a landmark for the junction between the transverse and sigmoid sinuses and, therefore, the transition between supratentorial space and posterior fossa. Moreover, beginning at the Ast and descending along the occipitomastoid suture, the occipital artery can be identified within the occipital groove. These landmarks can be utilized during the harvesting of the occipital artery for bypass surgery or simply to facilitate vascular control of the regional extracranial circulation.

Prominences and depressions

The nasion (Na) is located at the midpoint of the frontonasal suture, at its intersection with the nasal suture. Na is visible as a midline depression area superior to the nose bridge and between the two eyes. The distance from Na to Br is about 12-14 cm, and Lm is 24-26 cm posterior to the Na (Figure 4A, Interactive Model 3) [1]. The Na is also positioned at the same axial level of the corpus callosum (CC), precisely 12.6 ± 0.44 cm anterior to the genu and 19.8 ± 0.82 cm anterior to the splenium [7].

The glabella (Gl) is the most anterior midline point of the frontal bone. The Gl is located just superior to the Na, over the smooth surface between the orbital rims (Figure 2A). The anteroposterior length of the skull is equal to the distance from the Gl to opisthocranion, which is around 17 cm in adults (Figure 4A) [11]. The Gl is also a landmark for the frontal sinus.

The vertex is the most superior point of the skull and is located in the midline above the superior sagittal sinus (SSS) and between the the Br and Lm.

The superior temporal line (STL) is a subtle ridge of bone that extends from the posterior edge of the zygomatic process to the lateral surface of the parietal bone (Figure 2B, Interactive Model 4). At the level of the coronal suture, it splits into a superior and an inferior temporal line, which serves as an attachment site for the temporalis fascia and muscle, respectively [9]. The STL has a close relationship with speech areas: it demarks the superior limit of the IFG and is an important landmark for the supramarginal and angular gyrus [7].

The stephanion (St) corresponds to the intersection between the STL and the coronal suture (Figures 2B, 3A). It lies around 8 cm lateral to Br (Interactive Model 3) [2]. The inferior frontal sulcus (IFS) and pre-central sulcus (PreCS) meeting point is located about 0.5 cm posterior to the St [8]. Along with the STL, St is found 6.4 cm anterior to the euryon (Eu), and hence, the supramarginal gyrus (Figure 4A). The St is located on the same coronal plane as Broca’s area (Brodmann's area 44-45).

The Eu is a palpable prominence, localized in the middle of the parietal tuberosity (Figures 2B, 2D, 3). It is positioned at the junction between the STL and a vertical line that ascends from the posterior aspect of the mastoid process passing through the meeting point of the squamous and parietomastoid sutures. The Eu is always posterior to the post-central sulcus (PoCS) and corresponds to the posterior aspect of the supramarginal gyrus (Brodmann’s area 40). The Eu is located 1-2 cm anterior to the sulcus of Jensen and 1-3 cm lateral to the intraparietal sulcus (IPS) (Interactive Model 5) [2].

**Video 5:** Volumetric model of a specimen with bilateral windows dissections, opening of the dura and annotations of the principal cortical points, and their representation on the skull surface.

The obelion is located on the sagittal suture 2.5 cm anterior to Lm. The obelion sits between the two foramina parietalia (Figure 2C) [9]. 

Opisthocranion (Oph) is the most posterior prominent point along the midline (Figure 2D, Interactive Model 2). This point is located on the occipital bone, and is identified 3-4 cm superior the In, 2-4 cm inferior Lm, and 12-14 cm posterior to Br (Figure 4A) [2,8]. The Oph is one of the main landmarks for posterior craniotomy focused on the occipital lobe since the calcarine fissure and the cuneus base (Brodmann’s area 17 - primary visual cortex area) are located at the same axial plane of this prominence.

The inion (In) is a palpable midline prominence given by the external occipital protuberance (Figures 2D, 3B, 4B). The In is situated above the torcular Herophili, which corresponds to the confluence of the sinuses. The In provides attachment to the medial portion of the trapezius muscle and the superior segment of the nuchal ligament. This CP indicates the location of the tentorium and, therefore, the beginning of the posterior fossa. The line between the In and the Ast corresponds to the transverse sinus position and can be used as an inferior landmark for supratentorial exposures [3].

The nuchal lines are four curved crests of the external surface of the occipital bone (Figure 4B). The highest nuchal line begins at the posterior curvature of the skull, extends from midline to the lambdoid sutures bilaterally, and is where the occipital muscles join the galea. The medial nuchal line is a vertical crest that runs from the In to the foramen magnum and gives attachment to the nuchal ligament and the medial portion of trapezius, semispinalis capitis, and rectus capitis posterior minor muscles. This nuchal line overlies the occipital sinus. The superior nuchal line extends from the In to the lambdoid sutures and is the external landmark for the transverse sinus. This line gives attachment to the occipitalis, splenius capitis, and sternocleidomastoid muscles. The inferior nuchal line is a prominence that begins at the midpoint of the medial nuchal line and curves laterally and downward. The muscles attached to this line are the rectus capitis posterior major, rectus capitis posterior minor, and obliquus capitis superior [9].

The opisthion is a midline point for the posterior edge of the foramen magnum (Figure 4B) [2]. The basion is the midpoint of the anterior edge of the foramen magnum (Figure 4B) [9]. The distance from the Br to the basion is the height of the skull (approximately 13.2 cm). These points are used in spinal surgery as landmarks of the foramen magnum and to measure the distance with the atlas.

Cortical surface

**Figure 5:**
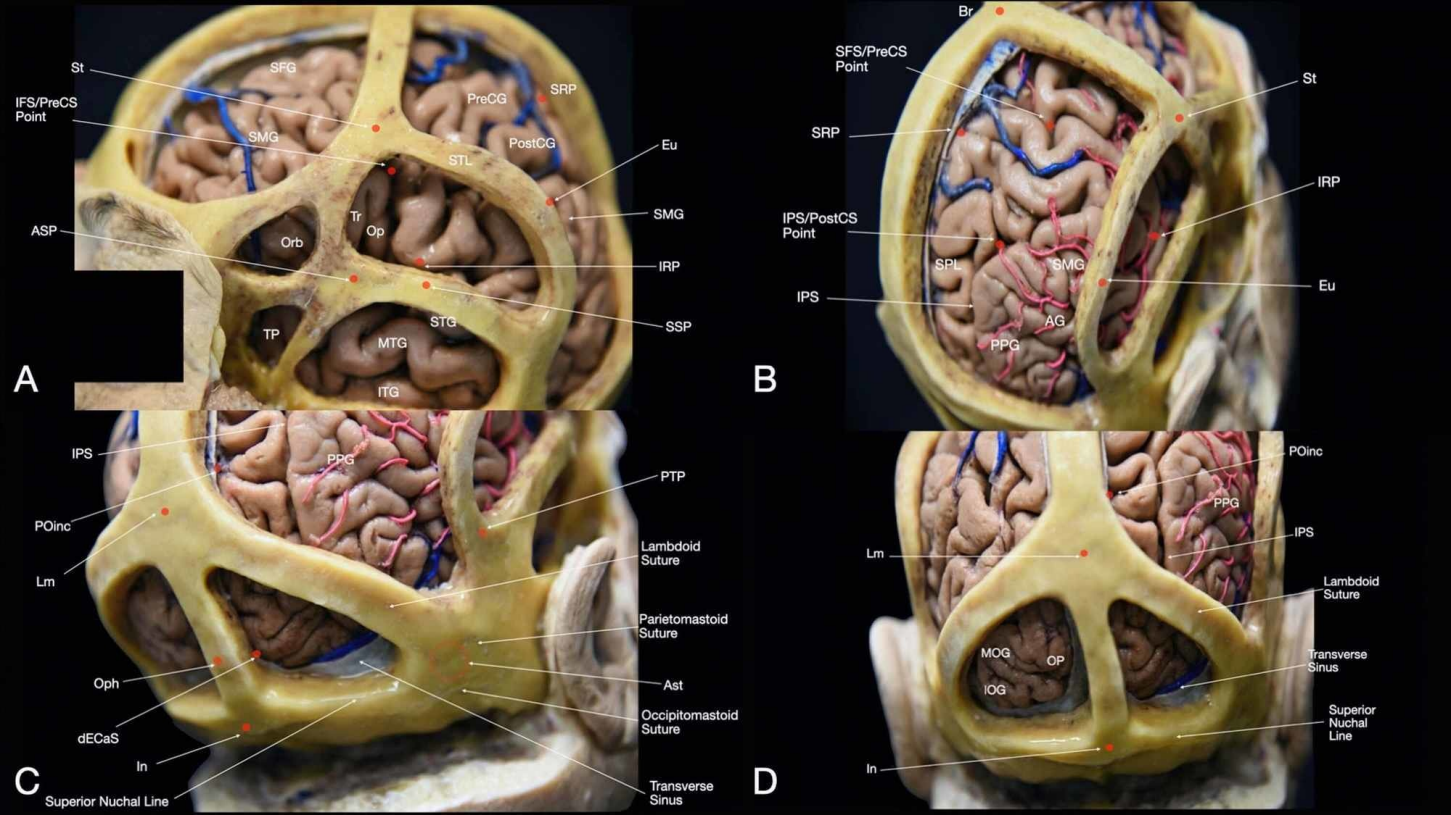
Relationship of relevant craniometric points and cortical surface structures. After performing the dissection of the superficial layer, seven bony windows were created outlining the main cranial landmarks. The correlation between the craniometric points and the brain surface is visible. (A) Lateral view of the head - superior frontal, inferior frontal, sphenoidal, superior parietal, inferior parietal, and temporal windows are shown; (B) superior-posterolateral view of the head - superior parietal, inferior parietal, and temporal windows are shown; (C) inferior-posterolateral view of the head - superior parietal, inferior parietal, temporal, and occipital windows are shown; (D) posterior view of the head - superior parietal and occipital windows are visible. AG: angular gyrus; ASP: anterior squamous point; Ast: asterion; Br: bregma; dECaS: distal extremity of the calcarine sulcus; Eu: euryon; IFS/PreCS point: inferior frontal sulcus/pre-central sulcus meeting point; In: inion; IOG: inferior occipital gyrus; IPS: intraparietal sulcus; IRP: inferior Rolandic point; ITG: inferior temporal gyrus; Lm: lambda; MFG: middle frontal gyrus; MOG: middle occipital gyrus; MTG: middle temporal gyrus; Op: pars opercularis of the IFG; Oph: ophistocranium; Orb: pars orbitalis of the inferior frontal gyrus (IFG); PoCG: post-central gyrus; POinc: parieto-occipital incisure point; PPG: posterior parietal gyrus; PreCG: pre-central gyrus; PTP: posterior temporal point; SFG: superior frontal gyrus; SFS/PreCS point: superior frontal sulcus/pre-central sulcus meeting point; SMG: supramarginal gyrus; SPL: superior parietal lobule; SSP: superior squamous point; SRP: superior Rolandic point; STG: superior temporal gyrus; STL: superior temporal line; St: stephanion; TP: temporal pole; Tr: pars triangularis of the IFG.

The ASyP marks the division between the proximal and distal portions of the SF. The ASyP can be identified on the cranial surface medial to the ASP (Figure 5A) [2]. This point is 2-2.5 cm anterior from the inferior Rolandic point (IRP).

The SF is the most prominent landmark of the lateral surface of the brain and can be divided into anterior and lateral segments [1]. The anterior portion of the SF starts at the anterior clinoid process extending laterally along the sphenoid ridge to reach the lateral convexity [12]. The ASyP corresponds to the anterior enlargement of the SF, where its anterior and lateral segments are divided. The lateral portion of the SF is formed by two anterior rami, horizontal and ascending, and a posterior ramus. The squamosal suture represents the direction of the lateral portion of the SF. The anterior horizontal ramus separates the pars orbitalis and triangularis of the IFG, whereas the anterior ascending ramus splits the pars triangularis and opercularis of the IFG. The posterior ramus divides the frontal and temporal lobe and runs obliquely, backward and upward to reach the inferior parietal lobule. Near the posterior ramus it is possible to identify a second enlargement of the SF, the posterior Sylvian point (PSyP), where two other rami arise. The posterior ascending branch ends inside the supramarginal gyrus and a distal descending branch which enters in the superior temporal gyrus [1]. During surgery, the ASyP and the PSyP are key landmarks for the splitting of the SF. Further medial dissection of the SF can lead to the operculoinsular, sphenoidal, and anterior basal compartments.

The IRP is the junction between the SF and the CS (Figures 5A, 5B). The superficial landmark to the IRP can be localized on the cranial surface at the intersection of the highest portion of the squamous suture with a vertical line that ascends from the preauricular depression, also known as the superior squamous point (SSP) [8]. The distance between the ASyP and the IRP is 2-2.5 cm. The IRP is also a landmark for the Heschl’s gyrus that is positioned in the superior temporal gyrus and, therefore, signals the beginning of Wernicke’s area (Interactive Model 5).

The IFS/PreCS point corresponds to the meeting of the IFS and the PreCS (Figure 5A). This point is commonly found underneath St. Specifically, the IFS is located an average distance of 0.17±0.5 cm superior to St and the preCS around 0.34±0.71 cm posterior to St [2]. The IFS/PreCS point is important to identify the pre-central gyrus (Brodmann’s area 4 - primary motor cortex) and delimitates the superior and posterior margin of the IFG.

The SRP is located at the intersection of the IHF and the CS (Figures 5A, 5B). The CP, which lies above the SRP, is about 5 cm posterior to the Br along the sagittal suture, and it is known as the superior sagittal point (SSagP) (Interactive Model 5) [8]. The SRP lies at the same coronal plane as the splenium of the corpus callosum and the quadrigeminal cistern [2].

The SFS/PreCS point marks the junction between the superior frontal sulcus (SFS) and PreCS (Figure 5B). This point can be localized in the skull 1.5 cm posterior to Br and 3 cm lateral to the sagittal suture, and it is called posterior coronal point (PCoP) (Interactive Model 5). The SFS/PreCS point is also an important landmark to identify the omega region (hand motor activation area, Brodmann’s area 6) in the pre-central gyrus. Moreover, it is coronally correlated to the thalamus and, hence, with the floor of the lateral ventricle (posterior to the foramen of Monro). The SFS/PreCS point is a crucial reference for superior frontal transulcal and interhemispheric transcallosal approaches to the ventricular cavity [1].

The IPS/PoCS point represents the connection between the IPS and the PoCS. This point can be identified on the skull as the intraparietal point (IPP) and is located 6 cm anterior to Lm and 5 cm lateral to the sagittal suture (Figure 5B, Interactive Model 3). The coronal projection of the IPS/PCS point corresponds to the atrium and trigone of the lateral ventricle. In surgery, it is a central landmark to confine the PoCS and as a starting point for a parietal transulcal approach to the atrium [1].

The parieto-occipital incisure (POinc) corresponds to the meeting point of the most superior point of the parieto-occipital sulcus and the external occipital fissure (EOF) into the medial aspect of each hemisphere (also called EOF medial point) [1,8]. The POinc lies underneath the lateral aspect of Lm (Figures 5C, 5D). This point is a surgical landmark for the posterior face of the precuneus along the IHF (Interactive Model 5).

The postSTS point is the most posterior portion of the superior temporal sulcus (STS), just before its trifurcation, where its middle and most horizontal branch penetrates into the angular gyrus [1]. This point lies underneath the posterior temporal point (PTP), which is positioned 3 cm above the ascending line from the meeting point between the parietomastoid and the squamosal sutures (Figure 5C, Interactive Model 3). The PSyP is approximately 2-3 cm anterior and superior to the postSTS. The PTP is useful in temporal posterior and inferior parietal craniotomies, and its axial projection corresponds to the atrium of the lateral ventricle.

## Discussion

Craniometric landmarks based on surgical applications

Craniometric Points for Ventricular Access

**Figure 6:**
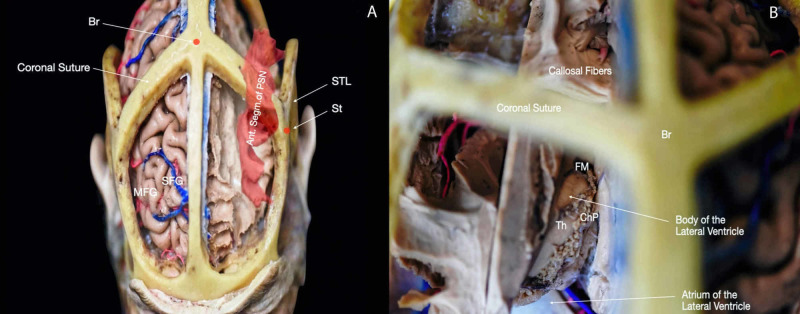
Subcortical structures and craniometric points. (A) Superior view of the head with windows craniotomies including white matter dissection showing the correlation of the anterior segment of the perisylvian network and the craniometric points; (B) superior view of the head with windows craniotomies with further with matter dissection showing the correlation of the body of the lateral ventricle and the craniometric points. Ant. segm.: anterior segment; Br: bregma; ChP: choroid plexus; FM: foramen of Monro; MFG: middle frontal gyrus; PSN: perisylvian network; SFG: superior frontal gyrus; STL: superior temporal line; St: stephanion; Th: thalamus.

Access to the ventricular cavities is one of the most performed procedures in neurosurgery, both in elective and emergency cases. The ventricles are localized in the center of the brain and, hence, reaching these cavities requires crossing through grey and white matter structures (Figure 6, Interactive Model 6). For these reasons, the cranial surface landmarks have been used to identify and describe burr hole locations to obtain safe pathways to reach different parts of the ventricles. Knowledge of the entry points is crucial to achieving an effective and secure ventriculostomy.

**Video 6:** Volumetric model of a specimen with bilateral windows dissections; on the left half head, the ventricular system is shown.

Kocher's point is the most utilized point for anterior access to the lateral ventricles. This point can be localized 11 cm posterior and superior to Na or 1-2 cm anterior to the coronal suture and 2-3 cm from the midline (Interactive Model 7). Kocher’s point is situated along the midpupillary line to avoid any disruption to the superficial venous system. The catheter should be inserted 6 cm below the skin surface, with a direction perpendicular to the meeting point between the ipsilateral medial canthus and external auditory meatus. Kocher’s point provides access to the frontal horn of the lateral ventricle. This precoronal point is so located to be lateral to the SSS and always anterior to the primary motor area [13].

**Video 7:** Composed volumetric model of a semitransparent skull depicting inner structures (i.e., left hemisphere and ventricular system) highlighting strategic burr holes based on craniometric points.

Keen’s point is located in the posterior parietal surface, 2-3 cm superior and posterior to the ear’s pinna. The catheter is direct cephalic and perpendicular to the temporal cortex (Interactive Model 7). The trigone of the ipsilateral ventricle is located 4-5 cm below [13].

Frazier’s point is another posterior parietal point. This point is positioned 6 cm superior to the In, and 3-4 cm lateral to the midline, above the lambdoid suture (Interactive Model 7). The catheter follows a medial and superior trajectory and is directed to a point placed 4 cm above the contralateral medial canthus. After 5 cm, the occipital horn and the body of the lateral ventricle should be reached [13].

Dandy’s point serves as an access point to the lateral ventricles from the posterior occipital region. The burr hole is placed 3 cm over the In and 2 cm laterally under the lambdoid suture (Interactive Model 7). The catheter is directed superiorly towards a point 2 cm above the Gl and inserted 4-5 cm to reach the occipital horn and body of the lateral ventricle [13]. 

Craniometric Points for the Identification of Cortical Areas

The primary motor cortex area is located at the pre-central gyrus on the dorsolateral surface of the brain (Interactive Model 1). Distance from the coronal suture to the CS measures around 5 cm. Localization of the CS and the motor cortex is a crucial aspect during frontoparietal craniotomies. Despite the common use of neuronavigation systems, a comprehensive understanding of the 3D topography of the primary motor cortex is commonly needed during cranial cases. Therefore, different surface topographic localization methods have been described to identify this critical area (Interactive Model 8) [14].

**Video 8:** Volumetric model of a specimen with bilateral windows dissections; on the left half head, the white matter dissection has been performed to show the segments of the perisylvian network.

**Figure 7:**
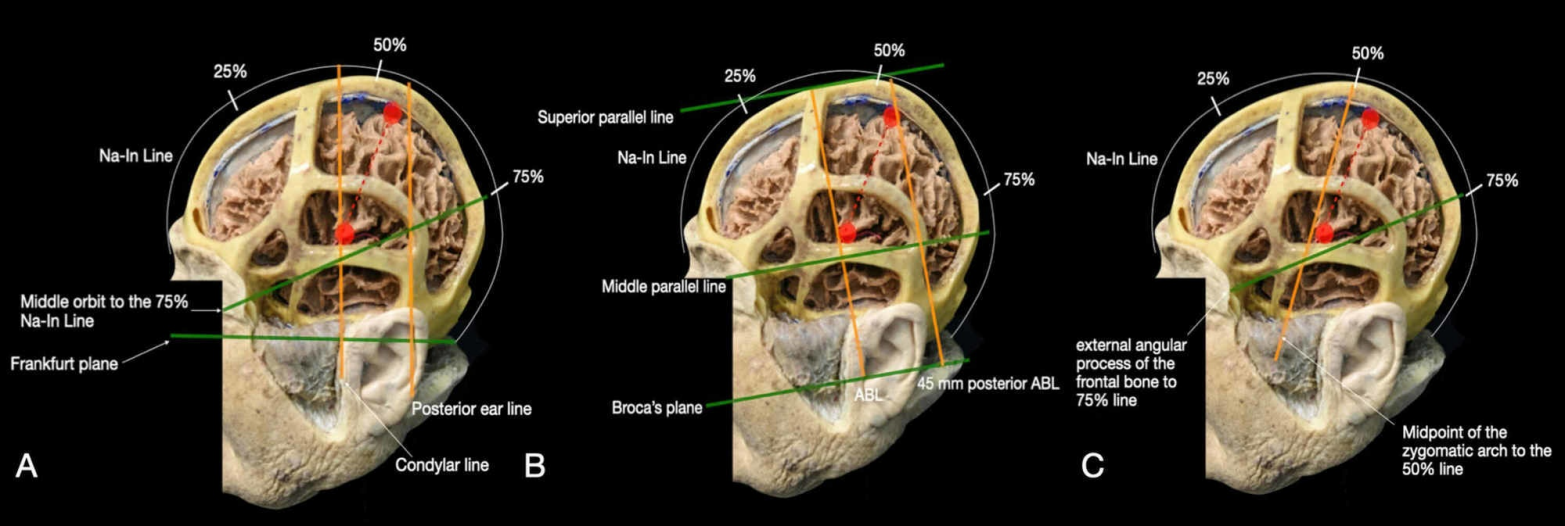
Representation of the main craniometric methods used to identify the central sulcus: (A) Taylor-Haughton method, (B) Broca’s method, and (C) Rhoton’s method. Red points and dashed lines represent the superior Rolandic point, inferior Rolandic point, and central sulcus. ABL: auriculobregmatic line; In: inion; Na: nasion.

The Taylor-Haughton method uses different lines and their intersection to identify the CS: the Frankfurt plane (i.e., a line that extends from the inferior margin of the orbit to the upper margin of the external auditory canal), the distance from Na to In along the calvarium (Na-In) divided in quarters (25%-50%-75%), the posterior ear line (i.e., a perpendicular line from the mastoid directed upward), condylar line (i.e., a perpendicular line from the mandibular condyle headed upward), the line from the middle of the orbit to the 75% mark along the Na-In, which corresponds to the SF from the orbit to the posterior ear line. The CS is situated 2 cm posterior to the 50% mark between Na-In (or the intersection between the Na-In and the posterior ear line), corresponding to the SRP, to the connection of the SF and condyle line, corresponding to the IRP (Figure 7A) [14].

Broca’s method uses as a reference Broca’s plane (BP), a horizontal line that extends from the base of the upper teeth through the bottom edge of the mastoid process. Two horizontal lines are then drawn parallel to BP: a superior line, intersecting with the Br, and a medial line from the external angular process of the frontal bone to just below the lambda suture. Then two vertical lines, perpendicular to BP, are drawn: the vertical auriculobregmatic line (ABL) and a second line 45 mm posterior to the ABL. The SRP can be located from the most superior point of the second vertical line at its intersection with the horizontal line intersecting Br. The IRP is situated at the intersection of the horizontal line from the external angular process of the frontal bone to the lambda suture and the ABL. The CS can be identified as the oblique line from the SRP and the IRP (Figure 7B) [14].

With Rothon’s method, the CS is detected with the use of three lines. The first is the Na-In line marked at the halfway point and at the three-fourths point (50% and 75%). The second one, which represents the SF, is an oblique line extending from external angular process of the frontal bone (i.e., anatomic keyhole) to the 75% mark of the Na-In line. The third line is an oblique line going from the midpoint of the zygomatic arch to the 50% mark of the Na-In line. The intersection of this last line with the 50% mark on the Na-In line corresponds to the SRP. The IRP is recognized at the junction of the midpoint of the third line with the second line (SF) (Figure 7C) [14].

Craniometric Points for the Identification of Vascular Structure

Awareness and identification of venous vascular structures of the brain surface are crucial during surgery to avoid early bleeding complications and to localize specific areas of interest. Therefore, some CPs have been detected to properly tailor the craniotomies (Interactive Model 9).

**Video 9:** Volumetric model of a hemisected skull with annotations showing the relationship between exocranial structures and critical venous structures.

**Figure 8:**
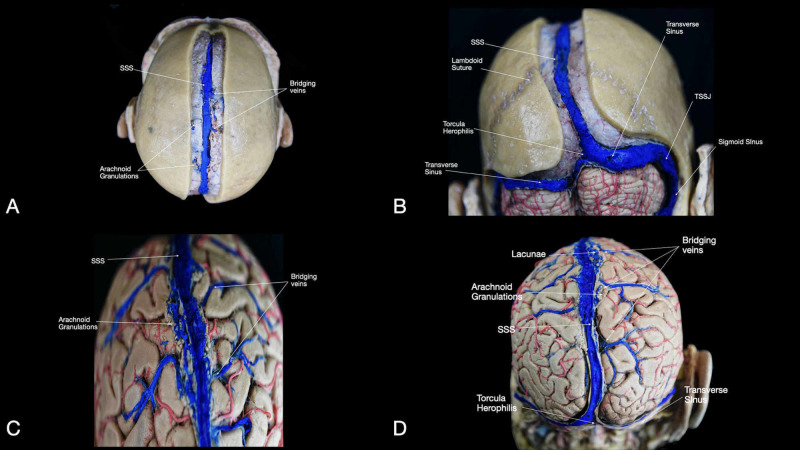
Overview of the major dural sinuses and veins of the brain. (A) Superior view of the head after removal of the superior sagittal suture and visualization of the SSS; (B) posterior view of the head after removal along the superior sagittal suture and opening of the posterior fossa, visualization of the SSS, torcula Herophili, transverse sinus, and sigmoid sinus; (C) visualization of the anterior portion of the brain underlying the venous structures (SSS, arachnoid granulations, and bridging veins); (D) visualization of the posterior portion of the brain underlying the venous structures (SSS, arachnoid granulations, bridging veins, torcula Herophili, transverse sinus). SSS: superior sagittal sinus; TSSJ: transverse-sigmoid sinus junction.

The SSS lies at the midline and runs from behind the frontal sinuses posteriorly in the shallow groove on the inner table of the cranium, below the sagittal suture, until the In, where it merges with the two transverse sinuses (Figure 8). The arachnoid granulations are commonly located 2.6 cm lateral to the SSS and can be found from 3.9 cm anterior to 7.3 cm posterior to the Br [7]. These granulations are important for the drainage of the superficial veins of the brain and the cerebral spinal fluid reabsorption into the venous system (Figures 8A, 8C, 8D) [7]. The bridging veins are the connection between the superficial venous system and the SSS. The connection between the superficial veins of the different lobes with the SSS can have different orientations, and they commonly run near the lateral wall of the SSS before draining into it. Accumulations of these veins are found conventionally between Br and L, predominantly in the right hemisphere: posteriorly to the frontal region above the genu of the CC and 4-6 cm from the torcular Herophili (Figures 8C, 8D). In this region also, the lacunae, enlarged venous spaces, can be found (Figure 8D). The lacunae are contained in the dura mater adjacent to the SSS and receive drainage of the meningeal veins. The cortical veins are known to pass beneath the lacunae to reach the sinus and, less frequently, to open into the lacunae. In the posterior frontal and parietal regions, the lacunae are the biggest and most consistent.

The vein of Trolard, also known as the superior anastomotic vein, is the largest anastomotic vein that crosses the cortical surface of the frontal and parietal lobes just between the SF and the SSS. The vein of Trolard is most frequently found in the PoCS lying 1.2 cm posterior to CS [15].

**Figure 9:**
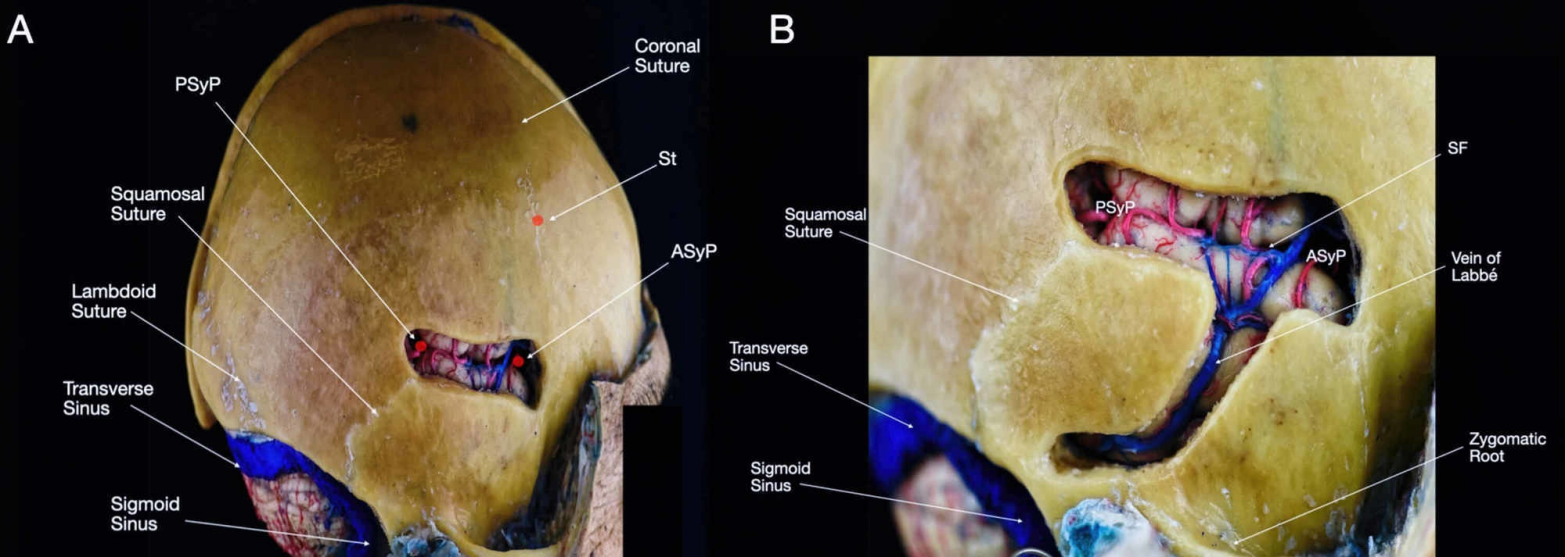
Overview of the SF and the vein of Labbé. (A) Lateral view of the head with exposure of the SF after selective drilling was performed over the central portion of the squamosal suture; (B) lateral view of the head with focus bone removal above the SF and the course of the vein of Labbé. ASyP: anterior Sylvian point; PSyp: posterior Sylvian point; SF: Sylvian fissure; St: stephanion.

The vein of Labbé, also known as the inferior anastomotic vein, arises from the middle portion of the SF and descends to the transverse sinus (Figure 9). This drainage is located 0.8-1.5 cm superior to the zygomatic arch and 2-5 cm posterior to the external auditory meatus opening. The vein of Labbé drains into the tentorial venous group, sigmoid or transverse sinus about 7 mm away from the superior petrosal sinus [15,16].

The transverse sinuses extend from the torcular Herophili to the sigmoid sinus bilaterally (Figures 8B, 8D, Interactive Model 9). They run laterally into a groove along the interior surface of the occipital bone, which can be identified externally by the line from In to Ast [1].

**Figure 10:**
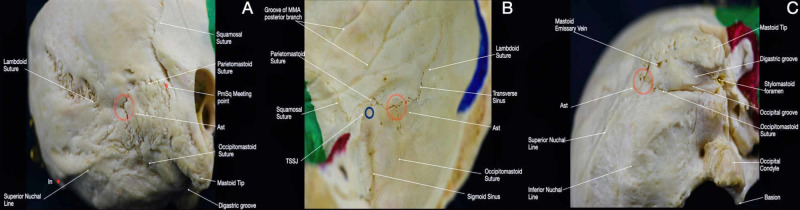
Overview of the Asterion and the transverse-sigmoid sinus junction. (A) Postero-lateral view of the skull; (B) interior view of the postero-lateral aspect of the skull; (C) inferior view of the postero-lateral aspect of the skull. Ast: asterion; TSSJ: transverse-sigmoid sinus junction.

The transverse-sigmoid junction is important for the placement of the strategic burr hole in posterolateral craniotomies. Traditionally, it has been associated with the Ast superficially, though it has been shown that the Ast overlays the transverse sinus more frequently [17,18]. Several methods to identify the transverse-sigmoid sinus junction have been described. The techniques that rely on local bony anatomy, observable in the surgical field, are the most accurate (Figure 10). Among these, the most accurate methods are the Ribas method, which recognizes the junction 1 cm anterior to Ast, with the superior edge of the burr hole adjacent to the petromastoid line, and the Teranishi method, which places the burr hole 0.65 cm inferior and 0.65 cm lateral to the Ast [17].

Craniometric Points for the Strategic Burr Hole Position

Sutures, cranial protuberances, and the CPs have been extensively used to locate critical structures while placing the burr holes of craniotomies. In this section, we will describe the main landmarks used in anterolateral cranial approaches (Interactive Model 7).

**Figure 11:**
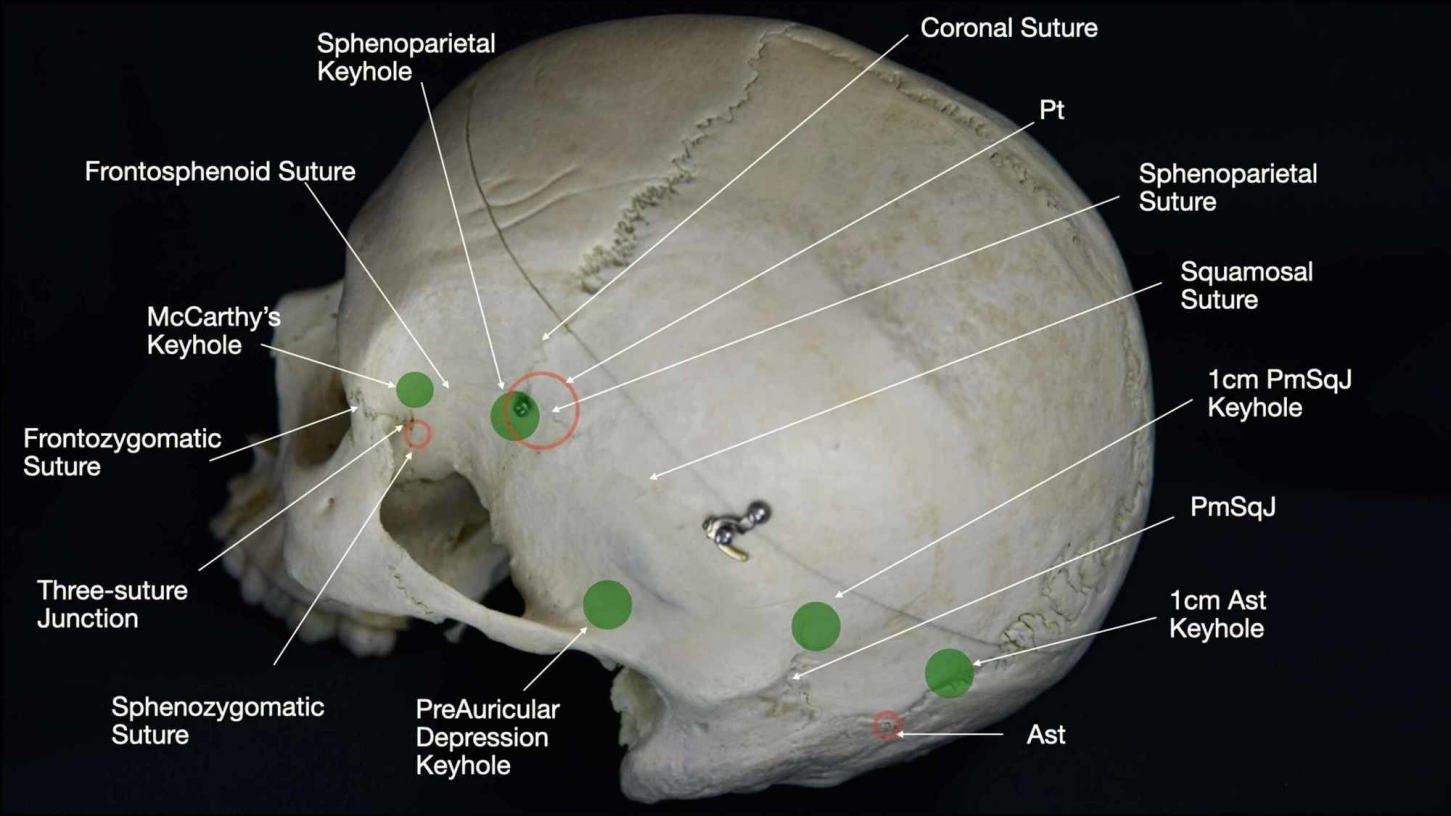
Representation of the strategic burr hole position (green spheres) on the lateral aspect of the skull. Ast: asterion; Pt: pterion; PmSqJ: parietomastoid-squamosal suture junction.

The MacCarthy keyhole is found in the frontal orbito-zygomatic region, most specifically on the frontosphenoidal suture. It is located approximately 6.8 mm superior and 4.5 mm posterior to the frontozygomatic suture [19]. This burr hole reveals frontal dura and the lower half’s area around the orbit, and it is used in the fronto-orbit-zygomatic craniotomies. In the pterional approach, the keyhole should be positioned along the frontosphenoid suture approximately 5-6 mm posterior to the junction of the frontosphenoid, frontozygomatic, and sphenozygomatic sutures: a landmark also referred to as the three-suture junction (Figure 11).

The sphenoparietal point corresponds to the projection of the most lateral and anterior aspects of the sphenoid ridge and the SF (Figure 11). This point constitutes an osseous transition between the anterior and middle fossae, which are the compartments that need to be exposed in frontotemporal approaches. The sphenoparietal point can be identified 21.72 mm posterior and 4.76 mm superior to the frontozygomatic suture [20].

The preauricular depression (PreAD) keypoint represents the most anterolateral position of the petrous bone, and thus the transition of the temporal fossa and the ascending petrous bone surface. This point is located above the PreAD, which is described as the ascendant portion of the superior margin of the posterior part of the zygomatic process, just anterior to the tragus and external acoustic meatus (Interactive Model 3). The burr hole performed on the PreAD keypoint exposes the posterior portion of the middle fossa. This point is related to the inferior temporal sulcus and in the coronal plane to the upper third of the clivus. The 1 cm above the external junction of the parietomastoid and squamous sutures (PMSQj) keyhole is a surgical landmark for the most posterolateral part of the intracranial petrous surface, which borders the petrous surface and the superior surface of the tentorium (Interactive Model 3). A burr hole placed at this point underlies the inferior temporal sulcus and, in the coronal plane, the posterior aspect of the midbrain. The preauricular point and the 1 cm above the PMSQj delimitate the external projection of the petrous bone; the middle fossa floor lies anterior to the first bur hole, and the superior surface of the tentorium lies posterior to the second bur hole. These two burr holes define the lateral aspect of the superior surface of the petrous bone and, therefore, the lateral aspect of the brainstem. The 1 cm above the Ast keyhole corresponds to the occipital notch, and it is related to the superior aspect of the transverse sinus and the superior tentorial surface (Figure 11, Interactive Model 7) [4].

## Conclusions

In this study, we review and describe all the crucial CPs that should be utilized in everyday practice. We aimed to facilitate the 3D comprehension of this critical landmark using VMs.

A thorough understanding of the relationship between the cranial surface and intracranial structures is paramount to avoid unnecessary exposure or damage to critical anatomical structures. Due to its superficiality, the correct interpretation of the neurovascular architecture underneath the craniotomy site is a primary and critical step in successful surgery. Although the average morphometrics found in the literature are not generally applied due to individual anatomical variations, the study of CPs still serves as a practical preoperative and intraoperative tool for the operator aiming to navigate the intricate neurosurgical anatomy. The knowledge of anatomical landmarks and a constant 3D visuospatial orientation of the relevant surgical topography remains an essential component to perform safe and efficient surgeries with successful outcomes.

## References

[1] Ribas GC (2018). Applied Cranial-Cerebral Anatomy.

[2] Ribas GC, Yasuda A, Ribas EC, Nishikuni K, Rodrigues AJ Jr (2006). Surgical anatomy of microneurosurgical sulcal key points. Neurosurgery.

[3] Ribas GC, Rhoton AL Jr, Cruz OR, Peace D (2005). Suboccipital burr holes and craniectomies. Neurosurg Focus.

[4] Ribas GC, Rodrigues AJ (2007). The suprapetrosal craniotomy. J Neurosurg.

[5] Rubio RR, Shehata J, Kournoutas I (2019). Construction of neuroanatomical volumetric models using 3D scanning techniques: technical note and applications. World Neurosurg.

[6] Jha RT, Magge SN, Keating RF (2018). Diagnosis and surgical options for craniosynostosis. In Principles of Neurological Surgery (4th Edition).

[7] Kendir S, Acar HI, Comert A, Ozdemir M, Kahilogullari G, Elhan A, Ugur HC (2019). Window anatomy for neurosurgical approaches. Laboratory investigation. J Neurosurg.

[8] Fernández-Cornejo V, González-López P, Abarca-Olivas J, Méndez-Román P, Moreno-López P, Sanchez del Campo F (2014). Craniometric points of the skull and the cerebral cortical surface. In 3D Neuroanatomy, Medical Atlas.

[9] Gray H, Warwick R, Williams PL (1973). Gray’s Anatomy (35th Edition). Gray’s Anatomy - 35th edition.

[10] Modasiya UP, Kanani SD (2018). Study of pterion and asterion in adult human skulls of north Gujarat region. Indian J Clin Anat Physiol.

[11] Kosif R, Sirmatel O, Canan A (2013). Morphometric measurements of the cranium in congenital bilateral blind males and females. Bosn J Basic Med Sci.

[12] Tanriover N, Rhoton AL Jr, Kawashima M, Ulm AJ, Yasuda A (2004). Microsurgical anatomy of the insula and the sylvian fissure. J Neurosurg.

[13] Morone PJ, Dewan MC, Zuckerman SL, Tubbs RS, Singer RJ (2020). Craniometrics and ventricular access: a review of Kocher's, Kaufman's, Paine's, Menovksy's, Tubbs', Keen's, Frazier's, Dandy's, and Sanchez's points. Oper Neurosurg (Hagerstown).

[14] Sun A, Hou LC, Cheshier SH, Sedrak M, Tse V (2014). The accuracy of topographical methods in determining central sulcus: a statistical correlation between modern imaging data and these historical predications. Cureus.

[15] Gusmão S, Reis C, Silveira RL (2001). Cranioencephalic relationships between Trolard and Labbé veins: neurosurgical applications [article in French]. Arq Neuropsiquiatr.

[16] Tubbs RS, Louis RG Jr, Song YB, Mortazavi M, Loukas M, Shoja MM, Cohen-Gadol AA (2012). External landmarks for identifying the drainage site of the vein of Labbé: application to neurosurgical procedures. Br J Neurosurg.

[17] Hall S, Peter Gan YC (2019). Anatomical localization of the transverse-sigmoid sinus junction: comparison of existing techniques. Surg Neurol Int.

[18] Tubbs RS, Loukas M, Shoja MM, Bellew MP, Cohen-Gadol AA (2009). Surface landmarks for the junction between the transverse and sigmoid sinuses: application of the "strategic" burr hole for suboccipital craniotomy. Neurosurgery.

[19] Rodriguez Rubio R, Chae R, Vigo V, Abla AA, McDermott M (2019). Immersive surgical anatomy of the pterional approach. Cureus.

[20] Reis BL, Silveira RLD, Gusmão SNS (2017). Sphenopterional point: strategic point for burr role placement in frontotemporal craniotomies. World Neurosurg.

